# Psychometric Properties of the 8-Item English Arthritis Self-Efficacy Scale in a Diverse Sample

**DOI:** 10.1155/2014/385256

**Published:** 2014-08-21

**Authors:** Sara Wilcox, Danielle E. Schoffman, Marsha Dowda, Patricia A. Sharpe

**Affiliations:** ^1^Prevention Research Center and Department of Exercise Science, Arnold School of Public Health, University of South Carolina, 921 Assembly Street, Columbia, SC 29208, USA; ^2^Department of Health Promotion, Education, and Behavior, Prevention Research Center, Arnold School of Public Health, University of South Carolina, 915 Greene Street, Columbia, SC 29208, USA; ^3^Department of Exercise Science, Arnold School of Public Health, University of South Carolina, 921 Assembly Street, Columbia, SC 29208, USA

## Abstract

Arthritis self-efficacy is important for successful disease management. This study examined psychometric properties of the 8-item English version of the Arthritis Self-Efficacy Scale (ASES-8) and differences in ASES-8 scores across sample subgroups. In 401 participants with self-reported doctor-diagnosed arthritis, exploratory factor analysis and tests of internal consistency were conducted. Concurrent validity was examined by associating ASES-8 scores with disease-specific, psychosocial, functional, and behavioral measures expected to be related to arthritis self-efficacy. All analyses were conducted for the full sample and within subgroups (gender, race, age, education, and weight status). Exploratory factor analysis for the entire sample and in all 12 subgroups demonstrated a one factor solution (factor loadings: 0.61 to 0.89). Internal consistency was high for measures of Cronbach's alpha (0.87 to 0.94), omega (0.87 to 0.93), and greatest lower bound (0.90 to 0.95). ASES-8 scores were significantly correlated with all measures assessed (*P* < 0.05), demonstrating concurrent validity. Those with a high school education or greater had higher ASES-8 scores than those with less than a high school education (*P* < .001); no other subgroup differences were found. The ASES-8 is a valid and reliable tool to measure arthritis self-efficacy efficiently and thereby reduce participant burden in research studies.

## 1. Introduction

Successful chronic disease management is contingent upon positive health behaviors, such as performing physical activity, adhering to appropriate medications, and eating a healthy diet. To help explain why certain people engage in healthier behavior than others, behavioral theories commonly incorporate self-efficacy or closely related constructs [1–5]. Self-efficacy is a person's confidence to perform a specific task or exhibit a specific behavior [1, 6]. Due to its importance in influencing health behaviors and health outcomes, many chronic disease self-management and other behavioral intervention studies [7–10], including those for people with arthritis [11–14], target self-efficacy and measure it as a study outcome.

Several scales are available to measure arthritis management self-efficacy. As part of the Stanford Arthritis Self-Management Study, the Arthritis Self-Efficacy Scale (ASES) was developed to be inclusive of all types of arthritis [15] and is widely used [16]. The ASES includes 20 questions that represent three subscales: pain, function, and other symptoms. Psychometric properties of the ASES and its three subscales are well established including high internal consistency, test-retest reliability, and validity [15]. In recent years, the ASES became available in a shortened, 8-item version (ASES-8) [17]. This 8-item version includes two items from the pain subscale, four items from the other symptoms subscale, and two new items that relate to preventing pain and fatigue from interfering with daily activities. Evaluations of the psychometric properties of the ASES-8 have only been reported for German [18] and Spanish [19] versions; the lack of psychometric studies of the English version is cited as a “major weakness” of the ASES-8 [16].

The purpose of this paper was to report the psychometric properties of the ASES-8 using a large and diverse sample of persons with arthritis. Specific goals were to (a) report the factor structure of the ASES-8, (b) assess the reliability (internal consistency) of the ASES-8, and (c) assess the concurrent validity of the ASES-8 by correlating it with measures for which the literature supports a relationship. These goals were applied to the sample as a whole as well as to subgroups according to gender, race, age, education, and weight status. This paper also examined whether ASES-8 scores differed according to gender, race, age, education, or weight status.

## 2. Materials and Methods

### 2.1. Procedure and Participants

Participants were recruited to take part in a randomized controlled trial that compared a self-directed exercise program to a self-directed nutrition program. Baseline data were used for this paper. Participants were recruited in a variety of ways with newspaper advertisements and worksite Listservs being the most successful. Potential participants were screened by telephone. Those who were at least 18 years old, had self-reported doctor-diagnosed arthritis, and experienced at least one symptom of arthritis (joint pain, stiffness, tenderness, decreased range of motion, redness and warmth, deformity, crackling/grating, or fatigue) were eligible for this study. Individuals were excluded if they had conditions that would be contraindications to physical activity or if they were already physically active.

Those who remained eligible and interested in the study participated in baseline measurement sessions. Prior to the scheduled measurement session, participants were mailed an informed consent form approved by the Institutional Review Board at the University of South Carolina and a survey. At the baseline measurement session, participants reviewed and signed the informed consent form, the survey was collected, and staff administered physical measurements and functional performance tests. Participants received a small cash incentive for taking part in the baseline measurement session.

### 2.2. Measures

*Demographic Variables. *Participants reported their gender, age, race, and education level. Age was categorized as 18–44, 45–64, and 65 years and older. Race was categorized as white or African American (3 participants with other races were not used in this subgroup analysis). Education level was categorized as college degree or less than a college degree.

*Body Mass Index.* Height to the nearest 0.25 inch and weight to the nearest 0.10 pound were measured by trained staff. BMI was computed as weight in kg divided by height in m^2^. BMI was categorized as normal (>18.5 and <25 kg/m^2^), overweight (25.0–29.9 kg/m^2^), or obese (≥30 kg/m^2^). 

*Arthritis Management Self-Efficacy.* The 8-item version of the Arthritis Self-Efficacy Scale (ASES-8) [17], based on the 20-item instrument [15], measured participants' confidence on a scale of 1 (very uncertain) to 10 (very certain) in their ability to manage symptoms of arthritis. Responses were averaged to yield a score ranging from 1 to 10 (higher = greater self-efficacy).

*Arthritis Symptoms. *Participants rated their arthritis symptoms in the past 2 weeks on a Visual Numeric Scale from 0 (no symptoms) to 10 (severe symptoms) [20]. Separate items evaluated pain, fatigue, and stiffness (higher = worse symptoms).

*Depressive Symptoms.* The 10-item Center for Epidemiological Studies Depression Scale (CES-D) [21, 22] measured depressive symptoms. On a scale of 0 (rarely or none of the time) to 3 (most or all of the time), participants rated the frequency with which they experienced 10 symptoms of depression during the past week. Responses were summed to yield a score ranging from 0 to 30 (higher = greater depressive symptoms). The measure had high internal consistency in this sample (Cronbach's alpha = 0.84, omega = 0.84, and greatest lower bound = 0.90).

*Self-Rated Health and Health-Related Quality of Life*. The Centers for Disease Control and Prevention's 4-Item Healthy Days Core Module measured health-related quality of life [23–26]. Participants rated their general health on a scale of 1 (poor) to 5 (excellent). They also reported the number of days (in the past 30 days) that their physical health was not good and their mental health was not good and the number of days that poor physical or mental health kept them from doing usual activities. Poor health days were summed to obtain a measure of health-related quality of life; a maximum score of 30 was assigned if the total number of poor health days reported exceeded 30. Thus, scores can range from 0 to 30 days (higher = poorer health-related quality of life).

*Disability.* The 20-item Health Assessment Questionnaire (HAQ) Disability Index measured self-reported disability [27–29]. On a scale of 0 (without any difficulty) to 3 (unable to do), participants reported the amount of difficulty they had in performing two or three specific activities in eight different categories (e.g., dressing, walking) over the past week. The total score was the mean of the eight categories (higher = greater disability). The measure had high internal consistency in this sample (Cronbach's alpha = 0.91, omega = 0.91, and greatest lower bound = 0.95).

*Functional Performance. *The 6-minute walk test measured functional exercise capacity [30, 31]. A 38-meter walking course was marked with cones in a level, carpeted hallway. Participants were instructed to walk as quickly as possible (not run) for 6 minutes. Assistive devices could be used. The score was the total distance walked (meters) in 6 minutes (higher = better functional capacity).

The GAITRite (CIR Systems, Havertown, PA), a portable walking mat with software, measured gait speed in meters/second [32, 33]. Participants were provided with sufficient distance to obtain their normal gait speed and then walked without shoes on the instrumented walkway. Assistive devices could be used. Participants completed three test trials, with the data averaged across the three trials. Gait speed is reported in meters per second (higher = faster).

The 30-second chair stand measured lower body strength [34, 35]. Participants sat in the middle of a chair with their back straight, feet flat on the floor, and hands on the opposite shoulder crossed at the wrist. Participants rose to a full stand and returned to a fully seated position, without using their arms. One practice of 1–3 repetitions was followed by one 30-second trial. The score is the total number of unassisted stands (higher = greater strength).

*Self-Reported Physical Activity.* The Community Health Activities Model Program for Seniors (CHAMPS) questionnaire, originally developed for older adults, is a 42-item self-report measure of the hours per week spent in light-, moderate-, and vigorous-intensity physical activity (MVPA) [36, 37]. A composite measure of total hours per week spent in all physical activities was computed.

### 2.3. Statistical Analyses

Analyses of internal consistency were conducted using R (Free Software Foundation, Inc., Boston, MA). All other analyses were conducted using SAS version 9.3 (SAS Institute, Cary, North Carolina). An exploratory factor analysis was conducted with responses to the ASES-8. The principal factor method was used. Four criteria were used to determine the number of factors: (a) eigenvalues had to be greater than 1.0 and had to explain at least 10% of the common variance, (b) visual examination of the screen plot was conducted to determine number of eigenvalues preceding the “elbow,” (c) item loadings had to exceed 0.40, and (d) the factor had to be interpretable. Consistent with recommendations by Peters [38] and to allow comparisons with other reliability reports in the literature, Cronbach's alpha, omega, and the greatest lower bound (GLB) were computed to assess internal consistency. Concurrent validity was assessed by testing associations between scores on the ASES-8 and variables expected to be related to the construct of arthritis self-efficacy. Pearson correlation coefficients were computed for arthritis symptoms (pain, fatigue, and stiffness), depressive symptoms, health-related quality of life, self-rated health, self-reported disability, functional performance measures (6-minute walk test, gait speed, and 30-second chair stand test), and participation in total physical activity. All analyses were also conducted separately for each sample subgroup according to gender, race, age group, education, and weight status. Finally, baseline differences in ASES-8 scores were examined according to gender, race, age group, education, and weight status. *T*-tests were used for dichotomous variables and ANOVA for categorical variables.

## 3. Results

### 3.1. Sample Characteristics

The study recruited 401 participants. Sample characteristics are shown in Table 1. Participants were mostly women (86%), white (64%) or African American (35%), obese (57%), between the ages of 44 and 64 years (66%), and college educated (61%).

### 3.2. Factor Structure

The exploratory factor analysis for the full sample indicated only one eigenvalue above 1.0 (5.09), and this result was visually confirmed on the scree plot. Each item in the ASES-8 loaded onto the single factor for the full sample. Factor loadings for each item were between 0.70 and 0.87 (see Table 2). The common variance explained by the single-factor solution was 64%.

Subgroup analyses (see Table 2) were also consistent with a one factor solution. For all study subgroups, eigenvalues on the first factor for all subgroups ranged from 4.59 to 5.74, common variance explained by the single-factor solution ranged from 57% to 72%, and factor loadings for each item were between 0.61 and 0.94. For three subgroups (white participants, participants of 18–44 years old, and participants with less than a college degree) the eigenvalues for the second factor exceeded 1.0 but were low (1.1, 1.0, and 1.1, resp.), although the explained common variance exceeded 10% (13% for each subgroup). However, no items loaded more heavily on factor 2 than factor 1, loadings on factor one exceeded the criteria of 0.40 in all cases, and the second factor was not easily interpreted. The scree plots for these three subgroups were similar to the overall sample scree plot. Therefore, a one factor solution best explained the data for all subgroups.

### 3.3. Reliability

Measures of internal consistency (see Table 2) were high for the full sample and for sample subgroups. Cronbach's alpha was 0.89 for the full sample (subgroup range: 0.87 to 0.94), omega was 0.90 (subgroup range: 0.87 to 0.93), and greatest lower bound was 0.92 (subgroup range: 0.90 to 0.95).

### 3.4. Concurrent Validity

As shown in Table 3, for the full sample, ASES-8 scores were significantly associated, in the predicted directions, with arthritis symptoms, depressive symptoms, health-related quality of life, self-rated health, self-reported disability, functional performance measures, and total physical activity. An identical pattern of associations was seen for all subgroups, although some of the correlations did not reach statistical significance.

Arthritis symptoms (pain, fatigue, and stiffness), depressive symptoms, more impaired health-related quality of life, and self-reported disability were negatively and significantly related to ASES-8 scores in all 12 subgroups. The magnitude of correlations ranged from *r* = −0.22 to *r* = −0.66. Self-rated health was positively and significantly associated with ASES-8 scores in every subgroup except men, and the magnitude of the significant correlations ranged from *r* = 0.27 to 0.50. The functional performance measures (6-minute walk, gait speed, and chair stands) were positively and significantly associated with ASES-8 scores in all but four subgroups (men, those of 18–45 years, those with less than a college degree, and those who were overweight), although the magnitude of the correlations was smaller than for the earlier described measures (*r* = 0.17 to 0.42). Finally, associations between total physical activity and ASES-8 scores were small but significant in six of the subgroups (*r* = 0.13 to *r* = 0.36).

### 3.5. Subgroup Differences in Arthritis Self-Efficacy

Scores on the ASES-8 were similar for men and women, *t*_(399)_ = 1.28, *P* = 0.20, and for whites versus African Americans, *t*_(250.71)_ = 0.79, *P* = 0.43. Scores did not differ according to weight status, *F*(2, 397) = 1.64, *P* = 0.19, or age group, *F*(2, 398) = 0.25, *P* = 0.78. Those with a college degree, however, had significantly higher scores than those with less than a college degree (6.64 ± 2.01 versus 5.79 ± 2.18, *t*_(399)_ = −3.99, *P* < 0.0001).

## 4. Discussion

Arthritis management self-efficacy, the belief in one's ability to manage the symptoms of arthritis, is a central component of chronic disease management. The purpose of this paper was to examine the factor structure, reliability, and validity of the ASES-8, a tool that can lessen respondent burden when compared to the original 20-item version. Our results support the use of the ASES-8 as a reliable and valid scale to measure arthritis self-efficacy.

For the sample as a whole, a one factor solution was found, with all items loading heavily on this factor. This same factor structure was seen for all subgroups. Although there was a second eigenvalue above 1.0 for 3 of the 12 subgroups (those who are whites, those of 18–45 years, and those without a college degree), the other criteria were not met for the second factor. Cronbach's alpha was also very high in the full sample and in all subgroups, consistent with reports of the German [18] and Spanish [19] ASES-8, as were the measures of omega and greatest lower bound. Associations between the ASES-8 scores and measures of arthritis symptoms, depressive symptoms, health-related quality of life, self-rated health, self-reported disability, functional performance measures, and total physical activity were statistically significant and in the anticipated direction for the full sample and for most subgroups. Finally, in another paper reporting the primary outcomes of our randomized trial, we report that ASES-8 scores improved significantly in response to both the exercise intervention and the nutrition control condition [39]. These findings are consistent with a large body of literature showing that arthritis self-efficacy is related to and predictive of meaningful physical and psychological health outcomes among adults with arthritis [14, 40–44], and the scale appears appropriate for a variety of sample subgroups.

There were several limitations to this study. Because the primary focus of the larger study was not to evaluate the psychometric properties of the scale, some subgroups had small sample sizes and may have been underpowered to detect associations. Samples sizes for men, those of 18–44 years, those of 65+ years, and those who were of normal weight were less than 100 individuals per subgroup. Women, whites, and those with a college degree were the most highly represented in the sample. Due to the use of secondary data, we were also unable to examine the test-retest reliability or divergent validity of the scale. We also recognize that an inherent limitation of survey measures of self-efficacy is that they may not be applicable to a given individual and may lack specificity when applied to an individual in treatment settings.

Furthermore, the cross-sectional study design does not allow us to make causal inferences. Those who report lower symptoms of arthritis, for example, may inherently feel more confident in their ability to manage arthritis than those with higher symptomatology. However, Arnstein and colleagues showed that self-efficacy mediated the relationship between pain intensity and disability among those with chronic pain and was a stronger mediator than was depression [45]. Future papers will examine the relationships between arthritis self-efficacy and study outcomes over time.

The primary strengths of the study include the large sample (*n* = 401) and the diversity along dimensions such as race, age, and arthritis type. Furthermore, a diverse set of measures were available for testing concurrent validity and included both self-report and objective measures and domains including arthritis symptoms, functional performance, health-related quality of life, and physical activity.

## 5. Conclusions

Despite the importance of arthritis management self-efficacy and the appeal of using an abbreviated measure, studies have not examined the psychometric properties of the ASES-8. This study demonstrated the factor structure, reliability, and concurrent validity of the scale for a diverse sample of adults with arthritis and in sociodemographic subgroups.

## Figures and Tables

**Table 1:** Sample characteristics (*N* = 401).

Characteristic	% (*n*)	Mean (SD)	Range
Gender, %			
Men	14.21 (57)		
Women	85.79 (344)		
Race, %			
White	64.00 (256)
African American	35.25 (141)
Other races or biracial	0.75 (3)		
Age group, %			
18–44 years	12.75 (51)		
45–64 years	65.50 (262)		
64+ years	21.75 (87)		
Educational attainment, %			
Less than college graduate	39.25 (157)		
College graduate	60.75 (243)		
Weight status, %			
Normal weight	14.50 (58)		
Overweight	28.50 (114)		
Obese	57.00 (228)		
Arthritis self-efficacy		6.32 (2.12)	0–10
Arthritis symptoms, %			
Pain		4.71 (2.32)	0–10
Fatigue		4.99 (2.65)	0–10
Stiffness		5.32 (2.55)	0–10
Depressive symptoms		6.47 (5.14)	0–28
HRQOL, days impaired in past month		10.42 (10.70)	0–30
Self-rated health (1 = poor; 5 = excellent)		3.07 (0.83)	1–5
Self-reported disability (3 = most disabled)		0.63 (0.52)	0–2
Functional performance			
6-minute walk, meters		494.05 (91.22)	151.46–721.57
Gait speed, meters/second		1.09 (0.22)	0.39–1.72
Chair stands, number in 30 seconds		9.99 (3.48)	0–24
Total PA, hours/week		9.94 (7.37)	0–46.75

Note: not all numbers sum to 401 due to missing data. HRQOL = health-related quality of life; min = minute; total PA = light-, moderate-, and vigorous-intensity physical activity.

**Table 2:** Factor loadings for one factor solution and reliability measures (Cronbach's *α*, omega, and greatest lower bound) for full sample and sample subgroups.

Item number	Full sample	Gender		Race		Age group			Educational attainment		Weight status		
		Women	Men	White∗	Af Am	18–45∗	46–64	65+	Not college grad∗	College grad	Normal	Overweight	Obese
1	0.72	0.74	0.61	0.69	0.79	0.61	0.75	0.71	0.74	0.70	0.56	0.76	0.75
2	0.78	0.80	0.62	0.73	0.85	0.67	0.80	0.80	0.77	0.78	0.73	0.80	0.79
3	0.87	0.87	0.85	0.84	0.91	0.89	0.85	0.90	0.88	0.86	0.88	0.84	0.88
4	0.86	0.86	0.83	0.83	0.89	0.89	0.83	0.91	0.87	0.84	0.85	0.88	0.84
5	0.87	0.87	0.83	0.84	0.89	0.87	0.86	0.88	0.89	0.84	0.85	0.89	0.85
6	0.70	0.69	0.75	0.65	0.74	0.69	0.69	0.71	0.72	0.65	0.73	0.74	0.67
7	0.84	0.85	0.73	0.80	0.88	0.84	0.84	0.82	0.84	0.83	0.87	0.85	0.82
8	0.74	0.73	0.79	0.70	0.77	0.75	0.74	0.71	0.71	0.74	0.71	0.72	0.75

*α*	0.89	0.90	0.89	0.87	0.94	0.91	0.92	0.89	0.91	0.91	0.88	0.93	0.92
Omega	0.90	0.90	0.89	0.87	0.94	0.91	0.92	0.90	0.91	0.91	0.88	0.93	0.92
GLB	0.92	0.93	0.93	0.90	0.96	0.95	0.95	0.92	0.94	0.94	0.94	0.95	0.94

Note: Af Am = African American. GLB = greatest lower bound. ∗A two-factor solution was obtained, but the second factor had a small eigenvalue (1.1 for whites, 1.0 for those of 18–45 years, and 1.1 for not college grad). Sample sizes were <100 for men, those of 18–44 years, those of 65+ years, and those who had a normal body weight. All items except item 7 started with the phrase “How certain are you that you can…” and ended with “decrease your pain quite a bit?” (1), “keep your arthritis or fibromyalgia pain from interfering with your sleep?” (2), “keep your arthritis or fibromyalgia pain from interfering with the things you want to do?” (3), “regulate your activity so as to be active without aggravating your arthritis or fibromyalgia?” (4), “keep the fatigue caused by your arthritis or fibromyalgia from interfering with the things you want to do?” (5), “do something to help yourself feel better if you are feeling blue?” (6), and “deal with the frustration of arthritis or fibromyalgia?” (8). Question 7 was worded as follows: “As compared with other people with arthritis or fibromyalgia like yours, how certain are you that you can manage pain during your daily activities?”

**Table 3:** Correlations between arthritis self-efficacy and health-related variables, overall and by sample subgroups.

Variable	Full sample	Gender		Race		Age group			Educational attainment		Weight status		
		Women	Men	White	Af Am	18–45	46–64	65+	Not college grad	College grad	Normal	Overweight	Obese
Pain	−0.39∗	−0.39∗	−0.43∗	−0.35∗	−0.44∗	−0.48∗	−0.42∗	−0.26∗	−0.32∗	−0.41∗	−0.39∗	−0.38∗	−0.40∗
Fatigue	−0.41∗	−0.42∗	−0.32∗	−0.41∗	−0.39∗	−0.35∗	−0.49∗	−0.23∗	−0.34∗	−0.44∗	−0.41∗	−0.38∗	−0.43∗
Stiffness	−0.39∗	−0.37∗	−0.53∗	−0.39∗	−0.38∗	−0.57∗	−0.41∗	−0.24∗	−0.32∗	−0.41∗	−0.47∗	−0.33∗	−0.41∗
Depressive symptoms	−0.43∗	−0.43∗	−0.47∗	−0.45∗	−0.42∗	−0.60∗	−0.45∗	−0.28∗	−0.40∗	−0.43∗	−0.52∗	−0.46∗	−0.38∗
HRQOL	−0.41∗	−0.38∗	−0.54∗	−0.43∗	−0.38∗	−0.35∗	−0.42∗	−0.40∗	−0.41∗	−0.38∗	−0.46∗	−0.47∗	−0.36∗
Self-rated health	0.32∗	0.36∗	0.07	0.31∗	0.33∗	0.46∗	0.30∗	0.29∗	0.33∗	0.29∗	0.50∗	0.32∗	0.27∗
Disability	−0.43∗	−0.41∗	−0.52∗	−0.38∗	−0.47∗	−0.66∗	−0.42∗	−0.33∗	−0.44∗	−0.37∗	−0.57∗	−0.22	−0.51∗
6-min walk	0.24∗	0.23∗	0.26	0.21∗	0.27∗	0.15	0.27∗	0.24∗	0.14	0.28∗	0.42∗	0.13	0.26∗
Gait speed	0.18∗	0.20∗	0.04	0.14∗	0.23∗	0.09	0.17∗	0.30∗	0.12	0.19∗	0.42∗	0.06	0.19∗
Chair stands	0.25∗	0.26∗	0.13	0.28∗	0.22∗	0.34∗	0.22∗	0.33∗	0.15	0.29∗	0.42∗	0.13	0.28∗
Total PA	0.15∗	0.16∗	0.12	0.14∗	0.13	0.25	0.10	0.23∗	0.14	0.13∗	0.36∗	0.11	0.13∗

Note: Af Am = African American; HRQOL = health-related quality of life (higher indicates more impairment); min = minute; total PA = light-, moderate-, and vigorous-intensity physical activity. Due to skewed distribution, a square root transformation was used for depressive symptoms, HRQOL, and total PA. ∗indicates *P* < 0.05.
